# Plasma Heme Oxygenase-1 Levels in Patients with Coronary and Peripheral Artery Diseases

**DOI:** 10.1155/2018/6138124

**Published:** 2018-08-07

**Authors:** Yoshimi Kishimoto, Susumu Ibe, Emi Saita, Kenji Sasaki, Hanako Niki, Kotaro Miura, Yukinori Ikegami, Reiko Ohmori, Kazuo Kondo, Yukihiko Momiyama

**Affiliations:** ^1^Endowed Research Department “Food for Health”, Ochanomizu University, Tokyo, Japan; ^2^Department of Cardiology, National Hospital Organization Tokyo Medical Center, Tokyo, Japan; ^3^Faculty of Regional Design, Utsunomiya University, Tochigi, Japan; ^4^Institute of Life Innovation Studies, Toyo University, Gunma, Japan

## Abstract

**Aims:**

Heme oxygenase-1 (HO-1) is an intracellular enzyme that catalyzes the oxidation of heme to generate CO, biliverdin, and iron. Since these products have antiatherogenic properties, HO-1 may play a protective role against the progression of atherosclerosis. However, plasma HO-1 levels in patients with atherosclerotic diseases, such as coronary artery disease (CAD) and peripheral artery disease (PAD), have not been clarified yet.

**Methods:**

We investigated plasma HO-1 levels by ELISA in 410 consecutive patients undergoing elective coronary angiography who also had an ankle-brachial index (ABI) test for PAD screening.

**Results:**

Of the 410 study patients, CAD was present in 225 patients (55%) (1-vessel (1-VD), *n* = 91; 2-vessel (2-VD), *n* = 66; 3-vessel disease (3-VD), *n* = 68). PAD (ABI < 0.9) was found in 36 (9%) patients. Plasma HO-1 levels did not differ between 225 patients with CAD and 185 without CAD (median 0.44 versus 0.35 ng/mL), but they were significantly lower in 36 patients with PAD than in 374 without PAD (0.27 versus 0.41 ng/mL, *P* < 0.02). After excluding the 36 patients with PAD, HO-1 levels were significantly higher in 192 patients with CAD than in 182 without CAD (0.45 versus 0.35 ng/mL, *P* < 0.05). HO-1 levels in 4 groups of CAD(−), 1-VD, 2-VD, and 3-VD were 0.35, 0.49, 0.44, and 0.44 ng/mL, respectively, and were highest in 1-VD (*P* < 0.05). In the multivariate analysis, HO-1 levels were inversely associated with PAD, whereas they were also associated with CAD. The odds ratios for PAD and CAD were 2.12 (95% CI = 1.03–4.37) and 0.65 (95% CI = 0.42–0.99) for the HO-1 level of <0.35 ng/mL, respectively.

**Conclusions:**

Plasma HO-1 levels were found to be low in patients with PAD, in contrast to high levels in patients with CAD.

## 1. Introduction

Heme oxygenase-1 (HO-1) is an intracellular enzyme that catalyzes the oxidation of heme to generate carbon monoxide (CO), biliverdin, and ferrous iron. These products have anti-inflammatory, antioxidant, antiapoptotic, and antithrombotic properties [1]. HO-1 is thus considered to have protective properties against the development of atherosclerosis, mainly due to the degradation of prooxidant heme, the generation of antioxidant biliverdin, and the production of vasodilator CO [2]. However, the HO-1 expression was observed throughout the development of atherosclerotic lesions from early fatty streaks to advanced lesions [3]. The HO-1 expression in endothelial cells, smooth muscle cells, and macrophages was upregulated upon the exposures to reactive oxygen species and oxidized LDL [4, 5]. In apoE-deficient mice, a lack of HO-1 accelerated atherosclerosis [6], whereas HO-1 induction reduced atherosclerosis in LDL receptor knockout mice [7] and Watanabe heritable hyperlipidemic rabbits [8]. Furthermore, the adenovirus-mediated gene transfer of HO-1 reduced atherosclerosis in apoE-deficient mice [9]. Therefore, HO-1 expression in atherosclerotic lesions is considered to be a protective response against the progression of atherosclerosis.

Recently, elevated blood levels of HO-1 were reported in some chronic diseases, such as type 2 diabetes mellitus (DM) and Parkinson's disease [10, 11]. Chen et al. [12] investigated HO-1 expression on blood leukocytes in patients with coronary artery disease (CAD). They showed HO-1 expression to be high in 30 patients with CAD. Although HO-1 is recognized to be released into the plasma from leukocytes, macrophage, smooth muscle cells, and endothelial cells that are activated or damaged by oxidative stress or inflammation [13, 14], few studies have examined plasma HO-1 levels in patients with atherosclerotic diseases. Idriss et al. [14] measured plasma HO-1 levels in 70 patients with stable CAD and 50 controls. They reported HO-1 levels to be higher in patients with CAD than in controls. In contrast, Signorelli et al. [15] reported plasma HO-1 levels to be lower in 27 patients with peripheral artery disease (PAD) than in 27 controls. Plasma HO-1 levels in patients with atherosclerotic diseases, such as CAD and PAD, remain unclear. Therefore, we examined the associations between plasma HO-1 levels and CAD and PAD in 410 patients undergoing elective coronary angiography who also had an ankle-brachial index (ABI) test to screen for PAD.

## 2. Methods

### 2.1. Study Patients

We investigated plasma HO-1 levels in 410 consecutive patients undergoing elective coronary angiography for suspected CAD at the Tokyo Medical Center who also had an ABI test to screen for PAD. The ABI was measured in a supine position after 5 mins of rest using the VaSera VS-1000 instrument (Fukuda Denshi, Tokyo, Japan), and PAD was defined as an ABI of <0.9 [16]. The severity of PAD was classified from stage I to IV according to Fontaine's classification. This study followed the methods of our previous study by Miyazaki et al. [17]. Any patients with a history of percutaneous coronary intervention or cardiac surgery were excluded. Patients with acute coronary syndrome, such as acute myocardial infarction (AMI) and unstable angina, were also excluded because HO-1 expression was shown to be upregulated in infarct myocardium [18] and because plasma and serum HO-1 levels were reported to be high in patients with AMI [14, 19]. Hypertension was defined as blood pressures of ≥140/90 mmHg or on drugs, and 249 (61%) patients were taking antihypertensive drugs. Hyperlipidemia was defined as an LDL cholesterol level of >140 mg/dL or on drugs, and 151 (37%) patients were taking statins. Diabetes mellitus (DM) (a fasting plasma glucose level of ≥126 mg/dL or on treatment) was present in 106 (26%) patients, and 158 (39%) were smokers (≥10 pack-years). Our study was approved by the institutional ethics committee of our hospital (R07-054/R15-056). After written informed consent was obtained, overnight-fasting blood samples were taken on the morning of the day when coronary angiography was performed.

### 2.2. Measurement of Plasma HO-1 Levels

Blood samples were collected in EDTA-containing tubes. Plasma was stored at −80°C. Plasma HO-1 levels were measured by an enzyme-linked immunosorbent assay (ELISA) with a commercially available kit (Human HO-1 ELISA Kit; Enzo Life Sciences Inc., Farmingdale, USA) at the Ochanomizu University in accordance with the manufacturer's instructions. The intra-assay and interassay coefficients of variation were all <10%.

### 2.3. Coronary Angiography

Coronary angiograms were recorded on a cineangiogram system (Philips Electronics Japan, Tokyo, Japan). CAD was defined as at least one coronary artery having >50% luminal diameter stenosis on angiograms. The severity of CAD was represented as the number of >50% stenotic vessels and the numbers of >50% and >25% stenotic segments. Coronary artery segments were defined as 29 segments according to the Coronary Artery Surgery Study (CASS) classification. All angiograms were evaluated by a single cardiologist (Y.M.), blinded to clinical and laboratory data.

### 2.4. Statistical Analysis

Any differences between two groups were evaluated by unpaired *t*-test for parametric variables, by Mann–Whitney *U* test for nonparametric variables, and by chi-squared test for categorical variables. Differences among three or more groups were evaluated by an analysis of variance with Scheffe's test for parametric variables, by Kruskal-Wallis test with Steel-Dwass test for nonparametric variables, and by chi-squared test for categorical variables. The correlation between HO-1 levels and the severity of CAD was evaluated by Spearman's rank correlation test. To determine the cut-off point of HO-1 levels for PAD, a relative cumulative frequency distribution curve was created, and then the optimum cut-off point was determined to be 0.35 ng/mL. A multiple logistic regression analysis was used to determine the independent associations between HO-1 levels and CAD or PAD. A *P* value of <0.05 was considered to be statistically significant. Results are presented as the mean ± SD or the median value.

## 3. Results

**Figure 1:**
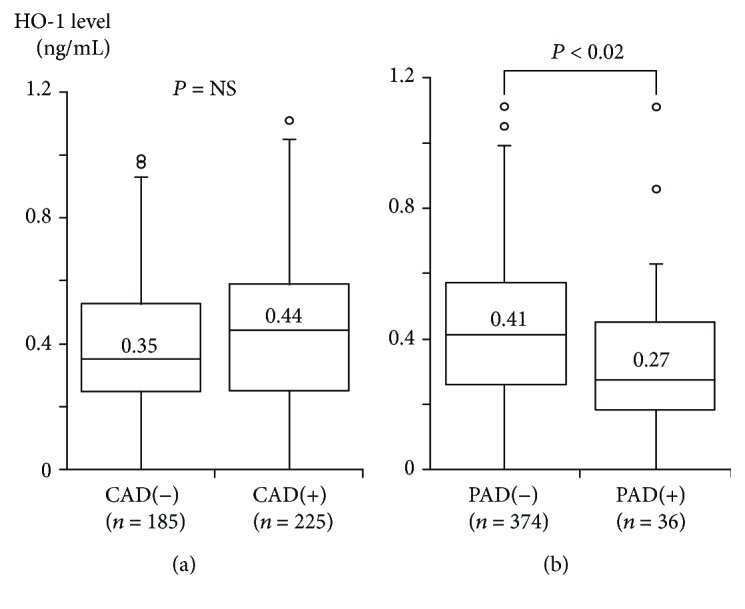
Plasma HO-1 levels and the presence of CAD or PAD. Plasma HO-1 levels tended to be higher in CAD than in CAD(−), but this difference did not reach statistical significance (a). In contrast, HO-1 levels were significantly lower in PAD than in PAD(−) (b). The central line represents the median, and the box represents the 25th to 75th percentiles. The whiskers represent the lowest and highest value in the 25th percentile minus 1.5 interquartile range (IQR) and 75th percentile plus 1.5 IQR, respectively.

Among the 410 study patients, CAD was present in 225 patients (55%) (1-vessel disease (1-VD), *n* = 91; 2-vessel disease (2-VD), *n* = 66; 3-vessel disease (3-VD), *n* = 68). Compared with 185 patients without CAD, 225 patients with CAD were older and had a male predominance and a higher prevalence of hypertension, hyperlipidemia, DM, and smoking (Table 1). Plasma HO-1 levels did not differ between patients with CAD and those without CAD (median 0.44 versus 0.35 ng/mL, *P* = NS) (Figure 1). The percentage of patients with an HO-1 level of <0.35 ng/mL were 40% and 49% in patients with CAD and those without CAD, respectively (*P* = NS). Furthermore, no significant difference was found in HO-1 levels among the 4 groups of CAD(−), 1-VD, 2-VD, and 3-VD (0.35, 0.45, 0.41, and 0.42 ng/mL). The Spearman's rank correlation test revealed no significant correlations between HO-1 levels and the numbers of >50% or >25% stenotic coronary segments (*P* = NS).

**Figure 2:**
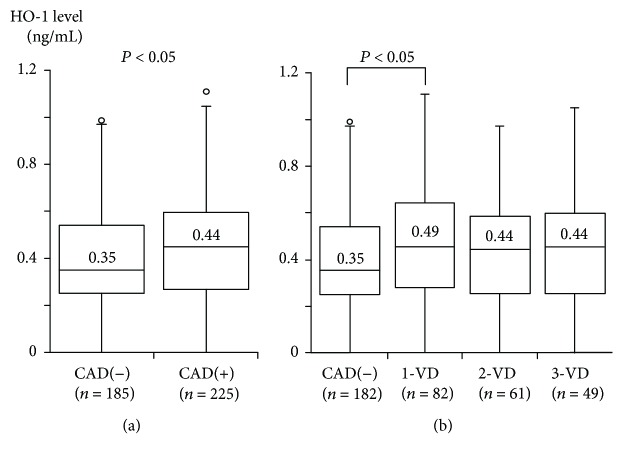
Plasma HO-1 levels and the presence of CAD or the number of stenotic coronary vessels among the 374 patients without PAD. After excluding 36 patients with PAD, HO-1 levels were significantly higher in CAD than in CAD(−) (a). Furthermore, HO-1 levels in 4 groups of CAD(−), 1-VD, 2-VD, and 3-VD were 0.35, 0.49, 0.44, and 0.44 ng/mL, respectively, and were highest in 1-VD (*P* < 0.05 by Kruskal-Wallis test) (b). The central line represents the median, and the box represents the 25th to 75th percentiles. The whiskers represent the lowest and highest value in the 25th percentile minus 1.5 IQR and 75th percentile plus 1.5 IQR, respectively.

Among the 410 study patients, PAD (ABI < 0.9) was found in 36 (9%) (3 of 185 (2%) patients without CAD and 33 of 225 (15%) patients with CAD). Compared with 374 patients without PAD, 36 with PAD were older and had a higher prevalence of hypertension, hyperlipidemia, and smoking (Table 2). Notably, plasma HO-1 levels were significantly lower in patients with PAD than in those without PAD (median 0.27 versus 0.41 ng/mL, *P* < 0.02) (Figure 1). Furthermore, patients with PAD more often had an HO-1 level of <0.35 ng/mL than those without PAD (61% versus 42%, *P* < 0.05). Of the 36 patients with PAD, 22 and 14 were found to have stage I and II PAD, respectively. HO-1 levels in patients with stage I and II PAD were 0.27 and 0.31 pg/mL, and the percentages of patients with <0.35 ng/mL were 63% and 57%, respectively. There were no significant differences in HO-1 levels between patients with stage I and II PAD. Interestingly, patients with PAD had a higher prevalence of CAD (92% versus 51%), especially 3-VD (53% versus 13%), than those without PAD (*P* < 0.001). After excluding the 36 patients with PAD, HO-1 levels were found to be significantly higher in 192 patients with CAD than in 182 without CAD (median 0.45 versus 0.35 ng/mL, *P* < 0.05) (Figure 2) and were less frequently <0.35 ng/mL in patients with CAD than in those without CAD (36% versus 49%, *P* < 0.025). Furthermore, HO-1 levels in the 4 groups of CAD(−), 1-VD, 2-VD, and 3-VD were 0.35, 0.49, 0.44, and 0.44 ng/mL, respectively, and HO-1 levels were highest in the 1-VD group (*P* < 0.05) (Figure 2). In particular, HO-1 levels in 1-VD were significantly higher than those in CAD(−) (*P* < 0.05).

HO-1 levels did not significantly correlate with age and LDL cholesterol and HDL cholesterol levels. As shown in Table 3, there were no significant differences in HO-1 levels between patients with and without hypertension, hyperlipidemia, statin use, DM, smoking, and aspirin use. To elucidate the independent associations between HO-1 levels and CAD or PAD, variables (age, gender, hypertension, hyperlipidemia, statin use, DM, smoking, and HO-1 levels) were entered into a multiple logistic regression model. HO-1 levels were found to be inversely associated with PAD, whereas they were also associated with CAD independent of atherosclerotic risk factors. The odds ratios for PAD and CAD were 2.12 (95% CI = 1.03–4.37, *P* < 0.05) and 0.65 (95% CI = 0.42–0.99, *P* < 0.05) for the HO-1 level of <0.35 ng/mL, respectively (Table 3).

## 4. Discussion

In the present study, plasma HO-1 levels did not differ between patients with CAD and those without CAD, whereas they were significantly lower in patients with PAD than in those without PAD. However, patients with PAD more often had CAD, especially 3-VD, than those without PAD. After excluding patients with PAD, HO-1 levels were significantly higher in patients with CAD than in those without CAD and were highest in 1-VD among the 4 groups of CAD(−), 1-VD, 2-VD, and 3-VD. In multivariate analysis, low plasma levels of HO-1 were found to be associated with the presence of PAD, in contrast to high HO-1 levels in patients with CAD.

HO-1 is an intracellular enzyme induced by oxidative stress and inflammation and is well-recognized to be one of the stress proteins [1, 13]. In macrophages, endothelial cells, and smooth muscles cells, HO-1 expression was shown to be upregulated by oxidized LDL [4, 5]. The HO-1 overexpression was reported in human atherosclerotic lesions [20]. Therefore, HO-1 overexpression in atherosclerotic lesions is considered to be a protective response against the progression of atherosclerosis. Since HO-1 is released into the plasma from leukocytes, macrophages, smooth muscle cells, and endothelial cells that are activated or damaged by oxidative stress or inflammation [13, 21], we hypothesized that plasma HO-1 levels would be high in patients with atherosclerotic diseases due to an adaptive response to oxidative stress-inflammatory repair process. Although the precise secretory pathway and the main source of HO-1 in plasma remain unclear, elevated plasma HO-1 levels were reported in patients with some chronic diseases, such as type 2 DM [10], as well as in patients with acute illness [22]. Regarding HO-1 in CAD, HO-1 expression in blood leukocytes was shown to be high in 30 patients with CAD [12]. Only one small study [14] reported plasma HO-1 levels to be higher in 70 patients with CAD than in 50 controls, but no significant difference was found in HO-1 levels among samples from peripheral vein, coronary sinus, and femoral artery. In our study, HO-1 levels did not differ between 225 patients with CAD and 185 without CAD. However, after excluding patients with PAD, HO-1 levels were significantly higher in patients with CAD than in those without CAD and were a significant factor associated with CAD. Thus, patients with CAD have high plasma levels of HO-1, probably reflecting a protective response against the progression of CAD. Furthermore, among the 4 groups of CAD(−), 1-VD, 2-VD, and 3-VD, plasma HO-1 levels were found to be highest in patients with 1-VD. Interestingly, the capacity to upregulate the HO-1 expression in leukocytes in response to oxidative stress was shown to be reduced in CAD patients with 2-VD or 3-VD [5]. Therefore, among the CAD patients, those with severe CAD, such as 2-VD or 3-VD, may have relatively lower plasma HO-1 levels and reduced protective response against oxidative stress compared with those with mild CAD, such as 1-VD. However, further studies are needed to show the association between plasma HO-1 levels and the progression of CAD in a prospective manner.

Atherosclerosis is a progressive disease that affects multiple vascular beds, and patients with CAD are often complicated by PAD [16, 23]. Our study was performed in 410 patients undergoing coronary angiography who also had an ABI test to screen for PAD. In our study, PAD was found in 3 of 185 patients without CAD (2%) and in 33 of 225 with CAD (15%). This prevalence of PAD was similar to that reported by Lee et al. [16] (4% of patients without CAD and 16% of those with CAD in 2543 patients undergoing coronary angiography). Regarding HO-1 levels in PAD, only one small study [15] investigated plasma HO-1 levels in 27 patients with PAD and 27 controls. They reported that HO-1 levels were lower in patients with PAD than in controls. In contrast to high HO-1 levels in CAD patients, especially those with 1-VD, we also found that plasma HO-1 levels were significantly lower in 36 patients with PAD than in 374 without PAD. As shown in Tables 1 and 2, patients with CAD or PAD were more often taking statin and aspirin. Statin and aspirin were shown to increase HO-1 expression in vitro [13]. However, in our study, no significant difference was found in plasma HO-1 levels between patients with and without statin or aspirin (Table 3). As a result, low HO-1 level was a significant factor associated with PAD independent of atherosclerotic risk factors. Thus, patients with PAD have low plasma levels of HO-1, suggesting that low plasma levels of HO-1 may be a marker reflecting the presence of PAD and may play a role in the development of PAD.

Patients with PAD usually have severe atherosclerosis in iliac and femoral arteries and often have CAD, especially 3-VD. Some differences in risk factors were reported between PAD and CAD [24, 25]. Although the mechanism of low plasma HO-1 levels in patients with PAD remains unclear, HO-1 defensive response to oxidative stress was reported to be attenuated at an advanced age [26] and the late stage of DM [27]. The HO-1 induction in plasma and tissues was decreased in aged rats after cardiac arrest [26]. In diabetic mice, HO-1 activity and mRNA expression were increased in the early stage of DM while decreased in the late stage of DM [27]. Therefore, the long duration of severe stress condition may cause some disruption of HO-1 defense system. Therapeutic angiogenesis for PAD by gene and cell therapy recently raised a great deal of hope for patients who cannot undergo revascularization. Because HO-1 has cytoprotective, antiapoptotic, and proangiogenic properties, HO-1 is a therapeutic target in PAD [28]. Gene and cell therapy with HO-1 were shown to be effective in animal models of limb ischemia [29, 30]. Since patients with PAD have low HO-1 levels in the blood, HO-1 may be used to treat patients with PAD to inhibit the progression of PAD.

Our study has several limitations. First, angiography was used to evaluate coronary atherosclerosis. Angiography cannot visualize plaques and only shows lumen characteristics. Second, PAD was found in 3 of 185 patients without CAD (2%) and in 33 of 225 with CAD (15%). This prevalence of PAD was similar to that reported by Lee et al. [16] (4% of patients without CAD and 16% of those with CAD). However, the small number of patients with PAD (*n* = 36) was a major limitation. Furthermore, in our study, an ABI test was used to screen for PAD, and PAD was defined as an ABI of <0.9 [16]. Angiography was not always performed to confirm the diagnosis of PAD. Third, HO-1 catalyzes the oxidation of heme to generate CO and biliverdin. These products have anti-inflammatory, antioxidant, antiapoptotic, and antithrombotic properties [1]. However, we could not measure blood levels of CO or biliverdin. This is one of the study limitations. Fourth, our study did not analyze any polymorphisms of HO-1 gene. Since some HO-1 gene polymorphisms were reported to be associated with CAD [31], these polymorphisms may have affected the plasma HO-1 levels in our patients with CAD and PAD. Fifth, our study was cross-sectional in nature, and it could not establish causality, since it only showed some associations and proposed some hypotheses. Finally, our study was on Japanese patients undergoing coronary angiography, who are generally considered to be a highly select population at high risk for CAD. Our results therefore may not be applicable to the general or other ethnic populations.

In conclusion, plasma HO-1 levels were found to be low in patients with PAD, in contrast to high levels in patients with CAD. Low HO-1 levels in patients with PAD may play a role in the development of PAD, whereas high HO-1 levels in patients with CAD, especially in those with 1-VD, may reflect a protective response against the progression of CAD.

## Figures and Tables

**Table 1:** Clinical characteristics and plasma HO-1 levels of patients with and without CAD.

	CAD(−) (*n* = 185)	*P* value CAD(−) versus CAD	CAD(+) (*n* = 225)	1-VD (*n* = 91)	2-VD (*n* = 66)	3-VD (*n* = 68)	*P* value among 4 groups
Age (years)	65 ± 12	<0.001	70 ± 9	68 ± 10	68 ± 10	73 ± 7	<0.001
Gender (male)	121 (65%)	<0.005	170 (76%)	68 (75%)	46 (70%)	56 (82%)	<0.025
Hypertension	116 (63%)	<0.001	180 (80%)	70 (77%)	52 (79%)	58 (85%)	<0.05
SBP (mmHg)	131 ± 21	NS	133 ± 21	133 ± 21	139 ± 21	130 ± 24	NS
Diabetes mellitus	25 (14%)	<0.001	81 (36%)	27 (30%)	26 (39%)	28 (41%)	<0.001
Smoking	59 (32%)	<0.01	99 (44%)	43 (47%)	28 (42%)	28 (41%)	<0.05
Hyperlipidemia	74 (40%)	<0.001	132 (59%)	50 (55%)	41 (62%)	41 (60%)	<0.001
Statin	46 (25%)	<0.001	105 (47%)	39 (43%)	31 (47%)	35 (51%)	<0.001
LDL-C (mg/dL)	112 ± 30	NS	114 ± 32	111 ± 34	117 ± 32	116 ± 29	NS
HDL-C (mg/dL)	59 ± 15	<0.001	51 ± 13	54 ± 14	49 ± 11	49 ± 12	<0.001
HO-1 levels	0.35	NS	0.44	0.45	0.41	0.43	NS
(ng/mL)	[0.25, 0.53]		[0.25, 0.59]	[0.27, 0.63]	[0.25, 0.56]	[0.19, 0.55]	
HO-1 < 0.35 ng/mL	91 (49%)	NS	89 (40%)	32 (35%)	27 (41%)	30 (44%)	NS
PAD (ABI < 0.9)	3 (2%)	<0.001	33 (15%)	9 (10%)	5 (8%)	19 (28%)	<0.001
Aspirin	59 (32%)	<0.001	130 (58%)	50 (55%)	44 (67%)	36 (53%)	<0.001

Data represent the mean ± SD or the number (%) of patients, with the exception of HO-1 level which is presented as the median value and interquartile range. SBP: systolic blood pressure; LDL-C: low-density lipoprotein cholesterol; and HDL-C: high-density lipoprotein cholesterol.

**Table 2:** Clinical characteristics and plasma HO-1 levels of patients with and without PAD.

	PAD(−) (*n* = 374)	PAD(+) (*n* = 36)	*P* value
Age (years)	67 ± 11	73 ± 9	<0.002
Gender (male)	264 (71%)	27 (75%)	NS
BMI (kg/m^2^)	23.7 ± 4.0	22.7 ± 3.2	NS
Hypertension	262 (70%)	34 (94%)	<0.005
Systolic blood pressure (mmHg)	132 ± 20	131 ± 31	NS
Diabetes mellitus	92 (25%)	14 (39%)	NS
Smoking	138 (37%)	20 (56%)	<0.05
Hyperlipidemia	181 (48%)	25 (69%)	<0.05
Statin	128 (34%)	23 (64%)	<0.001
LDL-cholesterol (mg/dL)	113 ± 31	115 ± 31	NS
HDL-cholesterol (mg/dL)	55 ± 15	48 ± 11	<0.01
HO-1 levels (ng/mL)	0.41 [0.26, 0.57]	0.27 [0.18, 0.45]	<0.02
HO-1 < 0.35 ng/mL	158 (42%)	22 (61%)	<0.05
CAD	192 (51%)	33 (92%)	<0.001
1-VD	82 (22%)	9 (25%)	NS
2-VD	61 (16%)	5 (14%)	NS
3-VD	48 (13%)	19 (53%)	<0.001
Aspirin	165 (44%)	24 (67%)	<0.025

Data represent the mean ± SD or the number (%) of patients, with the exception of HO-1 level which is presented as the median value and interquartile range.

**Table 3:** Associations between plasma HO-1 levels and atherosclerotic risk factors and medication.

	HO-1 levels	(ng/mL)	(+) versus (−) *P* value
*Hypertension*			
Hypertension (+) (*n* = 296)	0.38	[0.25–0.56]	NS
Hypertension (−) (*n* = 114)	0.44	[0.27–0.57]	
*Hyperlipidemia*			
Hyperlipidemia (+) (*n* = 206)	0.41	[0.25–0.54]	NS
Hyperlipidemia (−) (*n* = 204)	0.39	[0.25–0.59]	
*Statin use*			
Statin (+) (*n* = 151)	0.42	[0.25–0.54]	NS
Statin (−) (*n* = 259)	0.39	[0.25–0.58]	
*Diabetes mellitus*			
DM (+) (*n* = 106)	0.41	[0.25–0.56]	NS
DM (−) (*n* = 304)	0.39	[0.26–0.57]	
*Smoking*			
Smoking (+) (*n* = 158)	0.41	[0.25–0.54]	NS
Smoking (−) (*n* = 252)	0.39	[0.25–0.58]	
*Aspirin use*			
Aspirin (+) (*n* = 188)	0.43	[0.27–0.59]	NS
Aspirin (−) (*n* = 222)	0.36	[0.24–0.55]	

HO-1 levels are presented as the median value and interquartile ranges.

**Table 4:** Factors associated with CAD or PAD (multiple logistic regression analysis of the 410 study patients).

	Odds ratio	(95% CI)	*P* value
*CAD*			
Age (per 10 yrs increase)	1.52	(1.24–1.85)	<0.001
Male gender	1.79	(1.11–2.88)	<0.02
Statin use	2.01	(1.27–3.17)	<0.005
Diabetes	2.92	(1.72–4.93)	<0.001
HO-1 level (<0.35 ng/mL)	0.65	(0.42–0.99)	<0.05
*PAD*			
Age (per 10 yrs increase)	1.79	(1.20–2.68)	<0.005
Statin use	2.41	(1.17–4.94)	<0.02
Smoking	2.50	(1.21–5.13)	<0.02
HO-1 level (<0.35 ng/mL)	2.12	(1.03–4.37)	<0.02

The dependent variables were the presence of CAD or PAD. The analysis included age, gender, hypertension, hyperlipidemia, statin use, diabetes, smoking, and HO-1 level (<0.35 ng/mL).

## Data Availability

The data used to support the findings of this study are available from the corresponding author upon request.

## Supplementary Materials

Supplementary Materials Plots of plasma HO-1 levels in patients with and without CAD or PAD. The left figure shows plots of plasma HO-1 levels in patients with and without CAD, and the right figure shows plots of HO-1 levels in those with and without PAD.
