# Supplementary material for: Plasma Heme Oxygenase-1 Levels in Patients with Coronary and Peripheral Artery Diseases
Source: Dis Markers. 2018 Aug 7;2018:6138124. doi: 10.1155/2018/6138124 (PMC6109503; doi:10.1155/2018/6138124)
Supplement: Supplementary Materials — Plots of plasma HO-1 levels in patients with and without CAD or PAD. The left figure shows plots of plasma HO-1 levels in patients with and without CAD, and the right figure shows plots of HO-1 levels in those with and without PAD. [file 6138124.f1.docx]

**
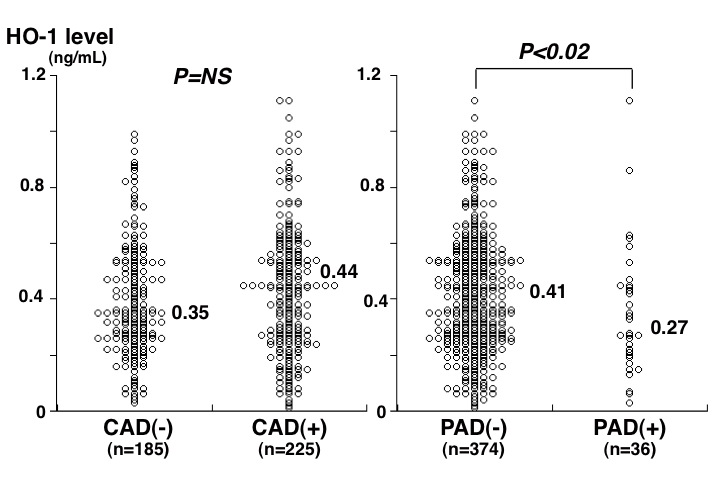
**

**Supplementary Figure. Plots of plasma HO-1 levels in patients with and without CAD or PAD.**

The left figure shows plots of plasma HO-1 levels in patients with and without CAD, and the right figure shows plots of HO-1 levels in those with and without PAD.
